# A Systematic Review of the Evolution of Laser Doppler Techniques in Burn Depth Assessment

**DOI:** 10.1155/2014/621792

**Published:** 2014-08-07

**Authors:** Manaf Khatib, Shehab Jabir, Edmund Fitzgerald O'Connor, Bruce Philp

**Affiliations:** St. Andrews Centre for Plastic Surgery and Burns, Broomfield Hospital, Chelmsford CM1 7ET, UK

## Abstract

*Aims*. The introduction of laser Doppler (LD) techniques to assess burn depth has revolutionized the treatment of burns of indeterminate depth. This paper will systematically review studies related to these two techniques and trace their evolution. At the same time we hope to highlight current controversies and areas where further research is necessary with regard to LD imaging (LDI) techniques. *Methods*. A systematic search for relevant literature was carried out on PubMed, Medline, EMBASE, and Google Scholar. Key search terms included the following: “Laser Doppler imaging,” “laser Doppler flow,” and “burn depth.” *Results*. A total of 53 studies were identified. Twenty-six studies which met the inclusion/exclusion criteria were included in the review. *Conclusions*. The numerous advantages of LDI over those of LD flowmetry have resulted in the former technique superseding the latter one. Despite the presence of alternative burn depth assessment techniques, LDI remains the most favoured. Various newer LDI machines with increasingly sophisticated methods of assessing burn depth have been introduced throughout the years. However, factors such as cost effectiveness, scanning of topographically inconsistent areas of the body, and skewing of results due to tattoos, peripheral vascular disease, and anaemia continue to be sighted as obstacles to LDI which require further research.

## 1. Introduction

Burn wounds that heal within a 3-week window have improved aesthetic and functional outcomes with a reduced degree of scarring [1]. This has meant that early accurate assessment of burn depth is essential in burn patients in order to decide between conservative treatment and surgical excision of the burn and grafting in order to achieve healing within this 2-3-week timeframe. Bedside clinical assessment is usually effective when the burns are either superficial or full thickness. However, in partial thickness burns where the burn depth is not well defined, clinical assessment is not as accurate. Overall, clinical assessment of burn depth when dealing with a burn of indeterminate depth has been shown to be accurate in only 65–70% of cases even when performed by an experienced burns surgeon [2]. For this reason a number of adjuncts to aid the clinician in making an accurate burn depth assessment were devised. Foremost among these techniques, and by far, the one that received unanimous approval by the burn community was laser Doppler technique to assess burn wound depth. Laser Doppler techniques utilize the Doppler effect described by the Austrian physicist Christian Doppler. In the case of laser Doppler techniques to assess burn depth, laser light is directed at moving blood cells in sampled tissue. The frequency change of the waves of laser light observed is proportional to the amount of perfusion in the tissue.

In this systematic review of the use of laser Doppler in assessing burn wounds we will trace the evolution of this technique and its application to burn depth assessment. Furthermore, the evidence for laser Doppler assessment will also be reviewed. Alternative techniques to determine burn depth will also be reviewed and compared to laser Doppler techniques. Finally, we intend to highlight current controversies and areas where further clarification and research are necessary.

## 2. Methods

Initially a study protocol was formulated with relevant inclusion and exclusion criteria defined for studies to be included in the systematic review (Table 1).

A literature search was then carried out on PubMed, Medline, Embase, and Google Scholar and the Cochrane databases from inception to February 2014 for studies on the topic of laser Doppler in burn depth assessment. The following key words were used: “laser Doppler imaging,” “laser Doppler flow,” and “burn depth.” The search terms were combined with the Boolean operator “and.” The references of selected studies were also perused for papers that may have been missed via the electronic search.

The title and abstract of all identified studies were examined by two reviewers (Manaf Khatib and Shehab Jabir). In cases where suitability of a study for inclusion in the review was unclear, the entire paper was obtained and assessed for suitability. Eligibility as mentioned above was determined by the criteria listed in Table 1. Any issues pertaining to eligibility of studies were solved via discussion with the senior author (Bruce Philp).

## 3. Results

A total of 53 studies were retrieved following the search. 27 studies were excluded following review of the title and abstract. The remaining 26 papers were reviewed to establish suitability for inclusion. The remaining 26 papers all met the inclusion criteria and were included in the review (Table 2).

## 4. Discussion

### 4.1. LD Flowmetry

Following Stern et al.'s proposal for the use of laser Doppler technology in burn depth assessment in 1975, a number of studies investigating and validating its use in clinical practice took place [3]. Green et al. published a landmark paper on this technology in 1988 and paved the way for forthcoming research [4]. The authors investigated the use of laser Doppler flowmetry on 13 burn wounds from 10 patients. Measurements were recorded twice daily after every dressing change in the first 72 h from the onset of the burn. Seven wounds healed conservatively within 21 days (healing group) and 6 wounds required excision and grafting (nonhealing group). The authors found statistically significant differences in laser Doppler measurements in the two groups (*P* < 0.02) at each 24 h interval measured. The authors did allude to several limitations in the study design, including; uncontrolled environmental factors and lack of knowledge of the effect of different dressings applied [4]. Despite the presence of limitations in the study and lack of description of the device and exact measurement of the laser Doppler values, the study was a pioneering study that instigated the development of further trials.

O'Reilly et al. soon followed the works of Green et al. and conducted a prospective cohort study in which they investigated the use of laser Doppler Flowmetry in 59 burns from 10 different patients [5]. LD assessment was compared to clinical assessment at initial presentation of the burn wound. Wounds deemed to require excision and grafting also underwent biopsies and histological assessment. LD values had no effect on the decision making of the burn surgeons and subsequent management. A cut-off point of 1.4 (arbitrary value of laser Doppler flow) was established and values above 1.4 had a 98.4% positive predictive value to heal within 21 days [5]. A substantial limitation to the study was that only burns that required surgery underwent biopsies and hence we have no way to determine the histological assessment of the wounds that healed conservatively [6]. This is especially important as the authors state that there was a “very poor correlation between LD values and the histologic depth in millimetres” [5]. The results obtained in view of the limitations do not support the strong conclusion of the authors that “LD flowmetry can diagnose accurately and early this critical level of thermal injury in burns of indeterminate depth” [5].

In another prospective cohort study by Waxman et al., 51 burn wounds from 33 patients were investigated [7]. Only patients with burns of indeterminate depth by clinical assessment and patients presenting within 48 h of the onset of burn were included in the study. The study not only investigated the accuracy of prediction of healing by LD flowmetry but also investigated the effect of different generated temperatures on the sensitivity and specificity of the assessment technique. The authors placed the measurement probe on different areas of burn wounds at temperatures of 35, 38, 41, and 44°C. All burns were managed conservatively, and burns that healed within 3 weeks were deemed as superficial partial thickness and burns that did not heal within this timeframe were deemed as deep dermal burns. 18 of the 51 burn wounds did not heal and required subsequent excision and grafting. The authors showed that burns with LD flow values of more than 6 mL/100 g/min at temperature of 35°C would heal in three weeks (100% specificity but poor sensitivity). Increasing the temperature to 44°C increased the sensitivity to 94% but decreased specificity [7]. A substantial limitation in the presentation of the result was that the authors failed to present the total body surface area (TBSA) of the burn wounds, as different sizes of burns will have different physiological consequences that could alter both core and peripheral surface temperatures.

Atiles et al. conducted a prospective cohort study that investigated 86 burn wounds from 21 different patients [8]. LD flowmetry was used with a contact probe heated to 39°C. Daily measurements were taken at days 0–3. Wounds were classified as either healed or not healed at 3 weeks after the burn. The study showed that burn wounds with more than 80 perfusion units (PU) will heal within 3 weeks with a sensitivity of 85%, specificity of 82%, positive predictive value (PPV) of 79%, and negative predictive value (NPV) of 87%. A PU of less than 40 predicted nonhealing at 3 weeks with a sensitivity of 46%, specificity of 100%, PPV of 100%, and NPV of 85% [8]. In the study there was no histological assessment to confirm that the nonhealing wounds were in fact deep at presentation. Confounding factors such as infection and cause of burn were not discussed and hence weakened the results of the study.

In a prospective cohort study by Park et al. in 1998, 100 burn wounds from 44 patients were investigated using LD flowmetry [10]. The primary outcome measure set by the authors was healing at 14 days. Only patients presenting within 72 h of injury were included. A value of more than 100 PU yielded a 90% PPV that the burn wound will heal within 14 days, and a value of <10 yielded a 100% PPV that the wound will not heal within 14 days. Values between 10 and 100 PU yielded a 96% PPV that healing will occur with scarring [10]. A criticism of the study is that the 14-day threshold to categorise burns into a healing and nonhealing group is not validated, the reason for choosing such threshold is not discussed and elaborated upon.

In a short report by Banwell et al., they used the same technique employed by Park et al. and found similar results and agreed that a 100 PU threshold was an accurate cut-off to predict wound healing [11]. They found good correlation between LD assessment and histological assessment. However, the authors discouraged the use of contact LD flowmetry due to the requirement of multiple measurements and contact with the burn wound. They shed some light on the Moor LDI device and touted it as a superior alternative to LD flowmetry due to the noncontact nature of measurement and the ability to cover a larger area [10]. Despite some bold conclusions in the report, there is no presentation of raw data or statistical analysis. However, this short report by Banwell et al. in 1999 set off the LDI revolution in motion and paved the way for the landmark paper on the use of LDI in burn depth assessment by Pape et al. (discussed below) in 2001.

Finally in 2003 Mileski et al. attempted to revive the use of LD flowmetry with a further study on the use of contact LD flowmetry in the assessment of burn wound depth [16]. Fifty six patients with 159 burn wounds were assessed. LD flowmetry was conducted daily from day 1 to 4 after burn. The results of the study showed 88% specificity and an 81% PPV for the identification of wounds that will not heal within 21 days [16]. The authors concluded what has already been established in the literature that LD assessment is more accurate than clinical assessment alone. However, by this time LDI had already superseded LD flowmetry and thus the results of this study added very little to this field.

The aforementioned studies all used LD flowmetry; this requires the direct contact of the laser Doppler probe to a burn wound which of course has inevitable negative implications, namely:

- patient comfort;
- need for patients to be still-implications in the paediatric population;
- infection and cross contamination due to contact of the instrument;
- small area of measurement and need for several readings to cover a burn wound.

### 4.2. LD Imaging

Niazi et al. were the first to study the noncontact laser Doppler imaging device in 1993 [9]. The authors studied 13 burn wounds that were scanned at 24, 48, and 72 h after injury. Only burns of indeterminate depth were included and all children were excluded. In contrast to LD flowmetry, the scans were conducted at a distance of 160 cm. LD assessment was compared to both clinical and histological assessment. They found a 100% correlation between LD assessment and histological assessment, compared to 70% correlation between LD assessment and clinical assessment and 40% correlation between clinical assessment and histological assessment [9]. The study did not include any statistical analysis and LD values were not explained and no cut-off point was defined.

**Figure 1:**
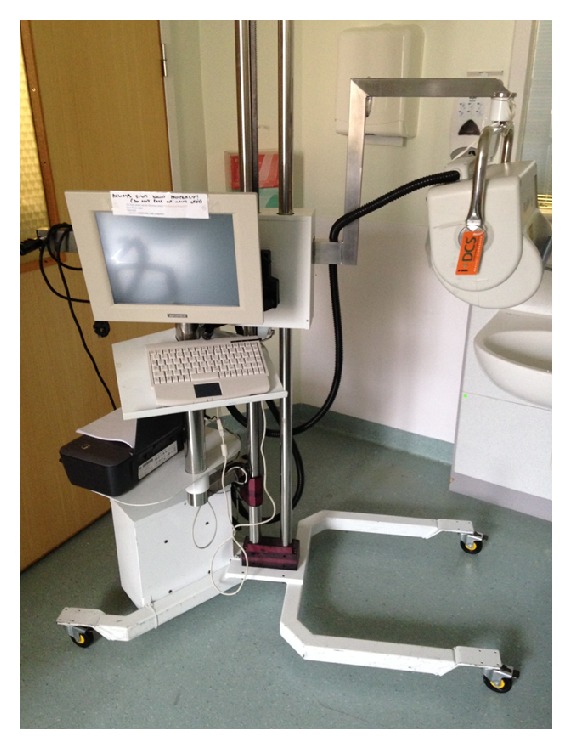
The Moor LDI system used by Pape et al. in their study and in our burns unit at St. Andrews Centre for Plastic Surgery and Burns.

**Figure 2:**
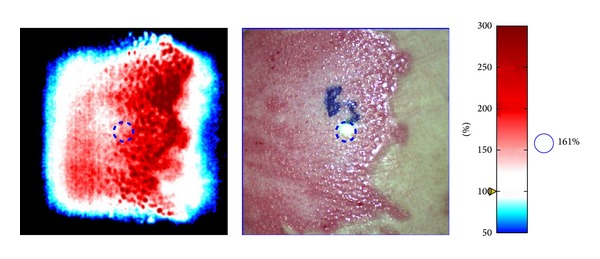
Appearance of a burn wound using the Aimago EasyLDI technology. It enables visualization of the microcirculation and the blood flow in small vessels with the increasingly red regions indicating greater blood flow.

In a prospective cohort study, Pape et al. assessed 76 wounds from 48 patients using LDI (Figures 1 and 2) [12]. They recorded LDI values between 48 and 72 h after injury and compared LD evaluation to both clinical assessment in all wounds and histological assessment in wounds that underwent surgery. Wounds that were deemed to be hyperperfused were managed conservatively with daily dressings and wounds deemed to be hypoperfused were managed surgically within the first 24 h of presentation. They found that the accuracy of LD assessment was 97% compared to 70% by clinical assessment [12]. It is imperative to mention that in 4 cases, the clinician ignored the judgement of the LD assessment, which judged the wound to heal within 21 days, and the cases were taken to theatre for excision and grafting. Histological assessment in those 4 wounds supported the clinical judgement. This illustrates that despite the high accuracy of LD assessment in this study, results should be correlated carefully with clinical judgement.

Kloppenberg et al. further assessed the use of LDI in burn depth assessment [13]. The authors studied 22 wounds from 16 patients. Only patients with a burn <10% TBSA were investigated in the study. The results of their study showed a 93.8% sensitivity and 100% specificity of day 4 after burn LD assessment prediction of healing within 21 days [13]. The results supported the new studies advocating the superiority of LD imaging over LD flowmetry.

In 2002, Holland et al. focused their study of LD assessment on the paediatric population only [14]. Critics of LDI have argued that accurate measurement of LD values in children will be difficult due to the need for the patient to remain still during the course of the assessment. The authors aimed to investigate if the results of their study on the paediatric population correlate with previous study findings in the adult population. 57 patients were studied over a 10 month period and patients were scanned 36–72 h after injury. They reviewed patients at 12 days to assess if wound healing has occurred or patients required surgery. At that time period, 17 of the patients required excision and grafting. In the deep dermal/full thickness cohort of patients, clinical examination and LDI assessment were 66% and 90% accurate, respectively. In the superficial partial thickness group the accuracy of clinical assessment was 71% and LD assessment was 96% [14]. The study represented an important landmark that proved the efficacy of this technique in the paediatric population despite the difficulties encountered with patient cooperation. A shortcoming of the study, however, is the 12-day threshold for determination of wound healing as it is a nonvalidated cut-off point and the authors do not elaborate on their choice.

Jeng et al. conducted a prospective blinded trial in 2003 [15]. The authors enrolled 23 patients with 41 different wounds of indeterminate depth. Daily assessment and decision of need for grafting were done by a clinician and recorded. LD scans were simultaneously conducted; however, the clinician remained blind to the LD assessment. The results of the study showed that clinical assessment agreed with LD assessment 56% of the time. In 21 wounds that were histologically analysed, burn depth assessment by clinicians was 71%. The authors further showed 100% agreement between histological analysis and LD assessment when wounds were hypoperfused. They calculated that LDI assessment would have saved a median of 2 days for every patient in determination for need of operating; this has some important implications on cost and reduced patient morbidity [15]. Despite an accurate assessment of need for grafting when the LD showed hypoperfusion, it is important to mention that 8/18 wounds that were deemed to be hyperperfused by LD assessment required grafting and deemed to be deep dermal or full thickness by histological assessment. The shortcoming raises some concerns and triggers the need for further assessment of LD thresholds for stratification of burn wounds.

In another prospective blinded trial, Riordan et al. studied 35 burn wounds from 22 patients using noncontact LDI [17]. The study focused on assessment of wounds to the upper and lower extremities. Scans were conducted at 48 h after the burn and all burn wounds had biopsies taken for histological assessment. A device-specific perfusion index showed a statistically significant inverse relationship between perfusion and burn depth. At a threshold of 1.3 perfusion index, LD assessment had 95% sensitivity and 94% specificity for prediction of wound healing at 21 days [17]. The sound methodology of the study yielded very positive results and further strengthened the argument for the use of LDI in assessment of burns of indeterminate depth.

La Hei et al. conducted another prospective blinded trial in a paediatric population [18]. 50 LD images from 31 patients were taken 72 h after injury. Two experienced burn surgeons were provided with clinical photographs of the wounds, relevant history, and LDI image. Another clinician blinded to the LDI values determined on regular intervals if a wound is healing or will require grafting. LD assessment showed a 97% correlation with clinical outcome. All wounds deemed to be deep by LDI did not heal within 21 days or required excision and grafting [18]. The study further dispels the concerns of the reliability of LD assessment in the paediatric population and strengthens the evidence for the use of LD assessment as an adjunct to clinical assessment.

In a comparison between two different modalities, McGill et al. assessed the benefit of videomicroscopy over LDI in the assessment of burn wound depth. Videomicroscopy (VM) was touted as a cheap alternative to LDI [19]. 27 wounds from 20 different patients presenting less than 72 h after a burn were assessed. LDI and VM assessments were carried out on all patients and the results were blinded to the clinical team. Three endpoints were established: healing within 21 days, early surgery, and delayed healing with need for grafting. VM assessment had had strong correlation with both LDI assessment and clinical outcome (*P* < 0.001). The authors concluded that VM is able to accurately assess burn depth and is comparative to LDI assessment with the advantage of being cheaper [19]. However, the results must be analysed with caution, as there was no histological assessment of the wounds that had early surgery and there is no way to find out if any of those wounds would have healed conservatively. Furthermore, VM assessment was carried out by an expert user and the results of the study may not be reproduced in other centres. VM assessment was also not tolerated well by children in the study. All the disadvantages of contact modalities in burn depth assessment still apply and hence its use over LDI is far-fetched.

In 2009, Hoeksema et al. aimed to identify the best day for LD assessment to be carried out and investigate at which day is LD assessment most accurate when being compared to clinical assessment [20]. In a prospective cohort study, the authors studied 40 burn wounds of intermediate depth. Both clinical and LD assessment were carried out in days 0, 1, 3, 5, and 8 after the burn. The two clinicians conducting the clinical assessment were blinded to the LD values. The outcome to compare to was healing within 21 days. For LD assessment the threshold for stratification of wounds in healing and nonhealing categories was 220 PU. On days 0, 1, 3, 5, and 8 LDI assessment was 54%, 79.5%, 95%, 97%, and 100% accurate, respectively. In clinical assessment it was 40.6%, 61.5%, 52.5%, 71.4%, and 100%, respectively. It was deemed that on day 3 LD assessment was significantly better than clinical assessment (*P* < 0.001) and also better on day 5 (*P* = 0.005) [20]. The study was the first to assess the relative benefit of LD assessment over clinical assessment on different days and provided important information to both clinicians in this field and for future research.

Cho et al. aimed to investigate a LDI cut-off that will allow prediction of healing and nonhealing at 14 days [21]. Patients less than 15 years of age with partial thickness wounds were recruited. LD scanning was conducted 48–72 h after the burn. Clinical assessment was conducted by two blinded clinicians. Healing was judged by observation of the wound on regular intervals for evidence of reepithelialisation. From the 181 wounds investigated, when using 250 PU as a cut-off point the sensitivity for healing within 14 days was 80.6% with a specificity of 76.9%. The mean PU for the healing group was 380 compared to 185 in the nonhealing group (*P* < 0.001) [21]. The lower sensitivity and specificity of LD assessment compared to previous studies, for prediction of healing within 14 days, illustrate the difficulty in predicting an outcome at such a short interval and the need for further research is necessary.

In another study focusing on the paediatric population, Mill et al. aimed to assess the validity of LD assessment in burn wounds in children [22]. A total 85 burns from 48 patients were investigated, time for wound healing and need for surgery were recorded. The different dressings used were also recorded. An important finding was that the use of Silver based dressings such as Acticoat did not interfere with the LD assessment. The use of another Silver based dressing, Silvazine, did however underestimate the perfusion in some wounds. The authors found congruence between the LDI colour palette of perfusion and the healing time [22]. A substantial limitation to the study is the lack of control over the time of scanning as the range of scanning was 0–120 h.

Kim et al. aimed to assess if LDI use helped in reducing the decision for operating on burn wounds [23]. A case-control trial was conducted, with patients undergoing LD scanning and clinical assessment (Group 1) and patients only being clinically assessed (Group 2). 196 patients were enrolled of which 49% underwent LD assessment. The mean time for decision to operate was 8.9 and 11.6 days in Groups 1 and 2, respectively, (*P* < 0.05) [23]. The reduction in decision to operate is inevitably beneficial in reducing length of stay, cost, and patient morbidity. The results of the study would have been more significant if randomisation was done; the reason for not randomising the population sample was not alluded to by the authors.

In a retrospective cohort study by Merz et al. they investigated the ability of LD flowmetry to accurately predict wound depth and healing potential in the first 24 h from the burn injury [24]. Twenty-eight patients with 173 wounds were retrospectively analysed. Regarding healing within 21 days, assessment at <24 h by LD flowmetry yielded a 93.1% accuracy when the values were >100 PU, and a value of <100 PU accurately predicated nonhealing in 88.2%. Further LD assessment at 3 and 6 days showed no significant reduction in perfusion [24]. The results are in disagreement Hoeksema et al.'s findings. The findings would have been strengthened if histological assessment was done on the 88 wounds that underwent surgery.

In a large prospective cohort study by Nguyen et al., 637 wounds from 400 patients were studied [25]. Paediatric patients were divided into two groups: presentation before (Group 1) and after (Group 2) 48 h. The sensitivity and specificity of LD assessment were 78% and 74% in Group 1, respectively. In Group 2, it was 75% and 85%, respectively. The difference was not statistically significant [25]. The findings support Merz et al.'s findings and illustrate the beneficial role of LD assessment in the acute phase.

Lindahl et al. operated the laser speckle imaging device in their study of 45 burns from 14 patients [26]. The speckle technology differs from the traditional laser Doppler technology. The device is composed of a source of laser and a detector camera. The emitted laser forms a speckle pattern once it contacts the skin; the contrast of the detected speckle image is affected by the underlying microcirculation and variation in flow. As opposed to LDI any artefact from movement is averaged out the speckle image [26]. The study showed a higher mean perfusion in wounds that healed in 14 days compared to wounds that did not heal. The difference between perfusion in those two groups was highest in 4–7 days after the injury [26].

In an interesting study by Menon et al., they investigated if the success of LD assessment in prediction of scald-burn healing potential is reproducible in friction burns [27]. A retrospective review was carried out on 36 friction burns. LD assessment accurately predicted wound healing in 64% of cases. The differences in the mechanism of burn were attributed to the lower accuracy compared to the literature [27].

Pape et al. conducted a large multicentre study that evaluated 433 burn wounds from 137 patients [28]. The aim of the study was to develop a validated colour code for LDI palette interpretation. They were able to correlate the colour palette with healing potential [28] as follows:

- healing within 14 days: red colour >600 PU;
- overlap area, healing within 21 days: pink colour 440–600 PU;
- healing between 14 and 21 days: yellow colour 260–440 PU;
- overlap area, healing most likely within 14–21 days: green colour 200–260 PU;
- healing >21 days: light blue colour <200 PU;
- nonhealing at 21 days: dark blue colour <140 PU.

In 2013, Park et al. aimed to analyse if LD assessment can expedite decisions regarding the need for excision and grafting in burn wounds of indeterminate depth [29]. A retrospective cohort study of 101 burn wounds was conducted. Patients were divided into a nonsurgical group (Group 1) and a surgical group (Group 2). There was a significant difference in mean PU between the groups (*P* < 0.001). A cut-off point of 154 PU yielded a sensitivity of 78.3% and 92.7% for prediction of need for surgery [29]. The results add further evidence for the support of LDI in burn depth assessment.

Finally, Stewart et al. conducted a prospective blinded control trial aiming to compare LDI assessment and clinical assessment in decision to operate on a burn wound [30]. The authors studied 105 burn wounds from 38 people. Using histological assessment as a gold standard, LD assessment was found to have a PPV of >90% [30]. The findings are in concurrence with previous studies aimed at assessing the benefit of LD assessment in prediction of need for surgery.

### 4.3. Alternative Techniques to Assess Burn Depth

#### 4.3.1. Fluorescein Dye

The use of dyes in the assessment of burn wound depth was first proposed by Lang and Boyd in 1942 [31]. As previously mentioned, in 1943 Dingwall studied the use of fluorescein dye to assess burn wound depth in animal models. He demonstrated that fluorescein would only reach areas with patent cutaneous circulation and thereby deeper burn areas can be marked [32]. However, the method was criticized due to the dynamic nature of a burn wound and the evolution of a burn in the first 24–48 hours [33]. The use of fluorescein dye was not adopted by many burn surgeons and the first study to be published in the literature that applied its use in human burns was not until 1961 [34]. Its use remained unpopular as quantifying the amount of dye in the circulation in certain parts of a burn was not possible before the invention of the fluorometer in the 1970s.

The fluorometer provided a method of quantification of fluorescein dye in the cutaneous circulation [35], and several studies investigated its use in both free flap monitoring [35–37] and burn wounds [38, 39]. Gatti et al. evaluated the ability of the fluorometer to distinguish partial thickness from full thickness burns after injection of fluorescein dye. They used this technique in 63 burn sites and showed that partial thickness burns exhibited the dye within 10 minutes of injection compared to full thickness burns where no dye penetrated the area [38]. Despite encouraging preliminary findings, the technique was regarded as cumbersome and nondefinitive in the assessment of burn depth. Black et al. assessed 59 and 37 burn sites in rats and human models, respectively, readings using a fluorometer were taken at different intervals. Actual depth of burn was judged by healing within 21 days. The results showed no significant difference of fluorescein uptake between partial and full thickness burns with large variability in both human and rat models [39].

Further research led to the discovery of indocyanine green (ICG) and its use in burns depth assessment. This was first described in 1992 by Green et al. who demonstrated the technique in a rat model [40]. They detected ICG fluorescence emission after administering intravenous ICG in partial and full thickness burns in rat skin. Different depths of burn were determined based on the intensity ratios compared to normal skin. An application of this technique in clinical practice was conducted by Still et al. in 2001 [41]. Fifteen burn wounds were assessed using the ICG method of assessment; fluorescence detected after intravenous injection of ICG correlated with the depth of burn as determined by biopsies from the burn sites and histological analysis. As expected, fluorescence was inversely related to burn depth. Cutaneous circulation and different degrees of brightness are demonstrated in the images. Another development in this field is the use of videoangiography to translate the fluorescence images into a colour-coded perfusion image indicating levels of tissue perfusion.

Despite some evidence supporting the use of ICG fluorometer, the method received criticism due to the extravasation of ICG dye in tissue which will inevitably render the method as inaccurate and yield false readings [42]. Moreover, reports of various side effects and anaphylaxis [43] and unknown safety of use in pregnant and breastfeeding women [44] has curtailed its use.

#### 4.3.2. Spectrophotometry

Spectrophotometry relies on the principle that partial thickness burn wounds still maintain their vasculature and capillary architecture whereas in full thickness burn wounds the blood vessels are thrombosed and damaged [34]. Anselmo and Zawacki were the first to describe the use of spectrophotometry in burn depth assessment [45], and infrared light was used to distinguish patent from thrombosed vessels and hence determine burn depth.

More recently, Tehrani et al. in 2008 used a noncontact spectrophotometry scope that uses polarised light from 400 to 1000 nm wavelengths. The scope detects any remitted light yielding images showing relative concentrations of haemoglobin, melanin, and collagen in a burn wound [46]. The authors compared the use of spectrophotometric intracutaneous analysis with LDI in the assessment of burn depth. Nine patients had their burn wounds imaged with both LDI and spectrophotometric techniques.

Results obtained from both modalities in the study were comparative and encouraging. Superficial burns had increased haemoglobin concentrations and lack of melanin compared to normal skin, whereby deep dermal burns had even higher concentrations of haemoglobin and a relative increase in melanin. Deriving absolute conclusions from the study is not possible though, due to the small number of burn wounds investigated.

#### 4.3.3. Thermography

Thermography is based on the principle that cutaneous circulation of a burn wound and hence temperature are inversely related to the depth of a burn wound [34]. In the 1960s, devices able to record differences in surface temperatures were used in monitoring the viability of flaps [47]. This was first applied to burn depth assessment by Mladick et al. in 1966 [48] and preliminary studies investigating its use found that the surface temperature of full thickness and partial thickness wounds differ by an average of 2 degrees Celsius [49]. In 1974, Hackett used this technique in assessing more than 100 burn wounds, yielding an accuracy of 90% [50]. Critics of this technique argue that temperature of a burn wound is a compound of various variable elements: room temperature, intravenous fluid resuscitation, blood flow, anatomical area, and other factors. Critics specifically argue that evaporative cooling will also lead to overestimation of burn wounds and hence inaccurate assessment and inappropriate management [51].

In 2005 Renkielska et al. investigated the correlation between static thermography and burn depth in an animal model [52]. They investigated the difference in temperature between a burn wound and an unaffected reference area of skin. Thermography was 93.8% accurate in predicting burn wounds that will heal in 21 days compared to 62.5% accuracy in the clinical method alone, this yielded a sensitivity of 97.7% and specificity of 85.8%. In a follow-up study by the same authors in 2006, they investigated the use of active dynamic thermography in burns depth assessment in an animal model. They studied 23 burn wounds of different depths that were inflicted on pigs. Comparing the method to clinical assessment, it had an accuracy of 100% in predicting burn wounds that will heal conservatively in 21 days compared to an accuracy of 61% via clinical assessment alone [53].

Hardwicke et al. recently investigated the role of high resolution digital thermal imaging in burns depth assessment. They studied 11 patients presenting with burns of different depths. Thermographic images were recorded 42 h and 5 days after a burn. They found that full thickness burns compared to normal skin are 2.3°C colder with strong statistical significance (*P* < 0.001), deep dermal burns were also found to be 1.2°C colder (*P* < 0.05), and superficial partial thickness burns were only 0.1°C colder [54]. This technique is presented as a safe, noncontact, inexpensive, and reliable adjunct in burns depth assessment that needs further evaluation and validation in large scale studies before drawing any solid conclusions.

This method of burn depth assessment relies on the principle that the more superficial a burn wound is the more present the dermal circulation is. This method allows a clinician to obtain close-up microscopic images of the underlying tissue and enable them to assess the presence or absence of blood vessels [44]. A contact near-infrared laser is applied on areas of concern and light reflected is captured and processed allowing visualisation of tissue planes up to 350 micrometers. In 2009, Altintas et al. used this method to assess 24 patients presenting with a burn. The wounds were investigated at 12, 36, and 72 h after the onset of a burn. After microscopy the burn wounds were divided into wounds predicted to heal within 3 weeks and wounds that will not heal within that timeframe. Several factors were assessed: presence of inflammatory cells, thickness of basal layer, and blood flow. Results showed an increase in blood flow in the group of burns that healed within 3 weeks compared to the nonhealing group at the different intervals measured. Moreover the thickness of the basal layer was preserved in the healing group compared to the obliteration of the basal layer in the nonhealing group at 36 h of measurement. The preliminary study showed some important findings and paved way for further research [55].

In 2011, Mihara et al. aimed to investigate the critical time for application of reflectance-mode confocal microscopy. This was an essential question to be answered due to the dynamic nature of a burn, and validation of the critical time for measurement is essential in preventing underestimation of burn wound depth and increased patient morbidity. They studied 41 patients with 44 different burn wounds. The use of videomicroscopy was compared to clinical assessment and showed a statistically significant superiority in accuracy of burn wound depth estimation (*P* = 0.001). The accuracy of videomicroscopy was found to be highest 24 h after onset of the burn injury [56].

Further research by the same authors in 2012 was conducted to develop a classification of burn depth and reliability of videomicroscopy. Forty-four patients with 56 burn wounds were investigated and results of videomicroscopy were compared to clinical outcome. This yielded an accuracy of 93% (sensitivity 81.8% and specificity 100%) [57]. Although research has showed positive results, opponents of this technique argue that the use of microscopy is cumbersome and painful to patients as contact between the wound surface and the scope is needed. Furthermore, due to the small surface area visualised by the scope, accurate measurement will require several measurements especially due to the heterogeneous nature of burn wounds [44]. Despite the limitations of its use, it remains an important adjunct that must be honed and developed to circumvent the criticisms received by this modality.

#### 4.3.4. Ultrasonography

Ultrasound techniques are used widely in both diagnostic and therapeutic techniques in different specialties. Goans et al. was the first to propose the notion of using ultrasound in assessing burn depth. The notion was based on the principle that ultrasound can detect the remaining dermal layer available above the subcutaneous tissue after a burn [58]. Preliminary studies in animal models showed that ultrasound techniques can be effective in determining which burn wounds will heal within 21 days and which will require excision and grafting [59, 60]. However, lack of translatable results in humans [61] coupled with limitations such as the need for contact with a burn and need for training in the interpretation of results deterred the acceptance of this modality in burn depth assessment [34].

Developments in the field of ultrasonography and the introduction of Doppler ultrasound led to further research in the field of burn depth assessment. In 2000, Seed et al. studied 78 burn wounds from 15 different patients. The noncontact Doppler ultrasound was used to visualise the different layers of skin within a burn wound: epidermis, dermis, and dermal-fat interface. Burns were deemed to be deep in nature if destruction of the dermal-fat interface is visualised. The accuracy of this method when compared to clinical outcome in the study was 96% [62]. Despite the promising findings, there is a lack of studies investigating the validity and reliability of this technique in burn depth assessment.

## 5. Conclusion

The need for an adjunct to clinical assessment of burn depth has instigated the development of a wide range of modalities aiming to improve our assessment of burn depth and patient care. It is clear from the discussion above that many of the other alternatives to LD techniques are either more cumbersome and more expensive or more difficult to adapt to the clinical setting resulting in LD techniques coming to the vanguard. Laser Doppler flowmetry and subsequently LDI has come to the forefront of technological adjuncts and several studies have illustrated the objective benefit of the use of LDI in conjunction with clinical assessment. The studies discussed have shown a significant improvement in prediction of burn healing and reduction of time for decision to operate when comparing LD assessment to clinical assessment only. The results indicate that the use of LD technology will reduce costs, length of stay, patient morbidity, and unnecessary surgery. Furthermore, studies with sound methodology have validated the optimal time for LD scanning.

From the available literature, it was apparent that studies did not agree on certain cut-off points of perfusion values. It is imperative for burn centres to validate the LD devices in use at their centres independently in order to find the most suitable cut-off points and levels of burn wound depth stratification.

Despite the positive results attained with the use of LDI, the studies in the literature have given rise to concerns that will need to be addressed in future technological developments and research projects. Opponents of the use of LDI technology argue that the commercial cost of the device [44] will render it unattainable to many burn units. This must drive further cost-benefit analyses to illustrate the potential cost saving of the technology. Moreover, the topographical artefacts that occur from scanning curved areas such as on flanks and extremities have challenged developers to innovate and design methods to circumvent such obstacles. Skewing of LD assessment results due to tattoos [63], presence of infection, and patient comorbidities such as peripheral vascular disease anaemia and patient use of systemic medication that may alter blood flow [44] have been shown in the literature. However, despite the shortcomings it must be stressed that LD assessment should contribute to the entire clinical picture and should be used as an aid rather than a replacement to clinical assessment.

An important point to shed light upon is the absence of any randomised controlled trials in this field. The paucity of randomised trials and absence of level I evidence in this field of research should drive large centres to conduct randomised studies and answer the research questions that arise regarding the use of LDI technology. In conjunction with the technological developments of the LD devices due to both clinical need and commercial competition, the plethora of research indicates that the age-old difficulty in assessing burn depth is a surmountable challenge. Developments in this field will inevitably lead to an improvement in clinical ability and ultimately patient care.

## Figures and Tables

**Table 1:** Inclusion and exclusion criteria for this systematic review.

Inclusion Criteria	
(i) Studies involving humans	
(ii) English language publication	
(iii) Studies published from inception of database to February 2014	
Exclusion Criteria	
(i) Use of LD techniques on animal models	
(ii) Non-English language publication	
(iii) Purely technical descriptions of the use of LD techniques with no analysis of outcomes	

**Table 2:** Summary of retrieved studies in the literature.

Authors country	Year	Type of study	Patient *n*	Burns *n*	Type of laser Doppler device	Surgery needed	Findings	Limitation
Green et al. [4], USA	1988	Observational Study	10	13	LD flowmetry Nonspecified type of LD scanner	6	Statistically significant difference in LD value between healing and nonhealing group	Lack of description of methodology of measurement

O'Reilly et al. [5], USA	1989	Prospective cohort *LD measurements did not influence clinical judgement *	41	59	LD flowmetry Laser flow blood perfusion monitor BPM403	8	LD < 1.4 PPV 98.4%, LD > 1.4 deemed superficial and will heal within 21 days	43 > 1.4 LD burned areas excised and grafted Day of measurement not specified

Waxman et al. [7], USA	1989	Prospective cohort *LDI within 48 *h* of burn* *Indeterminate depth only *	33	51	LD Flowmetry Laser flow blood perfusion monitor BPM403	18	100% specificity re: healing if flow >6 mL/100 g/min NPV 75%	TBSA not specified

Niazi et al. [9], UK	1993	Prospective cohort *Burns of indeterminate depth* *Children excluded *	13	13	LD imaging Newcastle laser Doppler scanner	7	Good correlation of LDI with histological assessment	No statistical analysis

Atiles et al. [8], USA	1995	Prospective cohort	21	86	LD flowmetry Perimed PF4000	33	<40 PU; Sen: 0.46, Spec: 1.0, PPV: 1.0, NPV: 0.85 >80 PU; Sen: 0.85, Spec: 0.82, PPV: 0.79, NPV: 0.87	No histological assessment. No burn cause identified

Park et al. [10], Korea	1998	Prospective cohort	44	100	LD flowmetry Periflux system 4001	Not specified	Primary outcome; healing at 2 weeks >100 PU 90% PPV 10–100 PU 96% PPV <10 100% PPV	Surgery not specified—just said not healed 2 weeks

Banwell et al. [11], UK	1999	Prospective cohort	30	n/a	LD flowmetry and LD imaging Moor LDI scanner	Not specified	Good correlation LDI results and histology	No stats

Pape et al. [12], UK	2001	Prospective cohort *Intermediate depth 48–72 *h* of presentation *	48	76	LD imaging Moor LDI scanner	25	97% PPV of LDI compared with 70% of clinical assessment	

Kloppenberg et al. [13], Netherlands	2001	Prospective cohort	16	22	LD imaging PIM 1.0 laser Doppler perfusion imager (Lisca development AB)	6	Sensitivity 100% and specificity 93.8% on day 4	Invalid statistical analysis

Holland et al. [14], Australia	2002	Prospective cohort *Paediatric burns only 12 days cut-off point for healing *	57	57	LD imaging Moor LDI V 3.1	17	Deep dermal; partial thickness Clinical examination 66% LDI 90%; clinical 71%, LDI 96%	Mobility of children No validated endpoint

Jeng et al. [15], USA	2003	Prospective blinded trial *Burns of indeterminate depth *	23	41	LD imaging Moor LDI-VR	7	56% agreement between clinician and LDI 71.4% accuracy of surgeon compared to histological diagnosis	8/18 burns deemed superficial by LDI but required grafting

Mileski et al. [16], USA	2003	Prospective cohort	56	159	LD flowmetry PF 4001 laser Doppler flowmeter	53	Sensitivity: 68% Specificity: 88% PPV: 81% NPV: 76%	Clinical assessment once versus serial LDI

Riordan et al. [17], USA	2003	Prospective blinded trial *Surgeon blinded to LDI result *	22	35	PIM #II LISCA	24	At threshold value of 1.3 Sensitivity: 95% Specificity: 94%	

La Hei et al. [18], Australia	2006	Prospective blinded trial *No clinical assessment done* *Assessment by images and LDI only *	31	50	LD imaging Moor LDI V2	22	Sensitivity: 97% Specificity: 100%	Statistical analysis and small number

McGill et al. [19], UK	2007	Prospective blinded comparison	20	27	LD imaging Moor LDI versus PW Allen videomicroscope: transcutaneous microscopy	10	LDI: sensitivity 100% VM: sensitivity for SPT 100%	No histological assessment Expert user of VM VM not tolerated by children

Hoeksema et al. [20], Belgium	2009	Prospective blinded trial *Early assessment of burns using LDI Intermediate depths * *Day 0*, *1*, *3*, *5*, *8*, and *21 *	40	40	LD imaging Moor LDI	12	Sensitivity increases with days after burn. Statistically significantly better than clinical assessment from day 3 Sensitivity: 100% Specificity: 92.3%	2 cases that required surgery and histology showed that burn wound was superficial in nature

Cho et al. [21], Republic of Korea	2009	Prospective cohort study *Paediatric burns Only burns of indeterminate depth * *48–72* h	103	181	LD imaging Periscan PIM 3	n/a	Healing by 14 days at PU of 250 Sensitivity 80.6% and Specificity 76.9%	No confirmation of superficial nature of burn with histology

Mill et al. [22], Australia	2009	Prospective cohort study *Paediatric burns Testing different effect of dressings *	48	85	LD imaging Moor LDI2	6	Scans within 24 h accurately predict outcome Colour palette corresponds to healing time. Cut-off of 14 days	No blinding Wide range of scanning time 0–120 h

Kim et al. [23], Australia	2010	Case-control trial *Only patients requiring grafting* <*16 years *	196	196	LD imaging Moor LDI2	196	Reduction in decision for surgery in LDI group 8.9 days versus 11.6 days in control group (*P* = 0.01)	No randomisation

Merz et al. [24], Germany	2010	Retrospective cohort study	28	173	LD flowmetry Laser Doppler O2C	88	Sensitivity: 80.6% Specificity: 88.2% PPV: 93.1% NPV: 69.8%	No histological assessment

Nguyen et al. [25], Australia	2010	Prospective cohort *Paediatric population Two groups; < and *>*48 *h* presentation *	400	637	LD imaging Moor LDI2-BI	89	<48 h Sensitivity: 78% Specificity: 74% >48 h Sensitivity: 75% Specificity 85%	No histological assessment in patients operated on

Lindahl et al. [26], Sweden	2013	Prospective cohort	14	45	LD imaging Laser Speckle contrast imager (Perimed AB)	n/a	Higher perfusion in burns healing in less than 14 days compared to more than 14 days from day 0 from burn.	Small sample of patients No gold standard to compare to

Menon et al. [27], Australia	2012	Retrospective cohort *Friction burns in paediatric population *	36	36	Not specified	12	64% accuracy of LDI predicting burn outcome	Small sample of patients No gold standard to compare to

Pape et al. [28], Multicentre	2012	Prospective cohort	137	433	LD Imaging Moor LDI	ns	Development of validated colour code for interpretation and link to burn outcome	

Park et al. [29], Korea	2013	Retrospective cohort	96	101	LD imaging Periscan PIM3 (Perimed AB)	46	Cut-off point of 154.7PU Sensitivity: 78.3 Specificity: 92.7	

Stewart et al. [30], Canada	2012	Prospective blinded control trial	38	105	LD imaging Moor LDI 2-B1	64	LDI has PPV > 90% accurate in determining need for grafting	
